# Mirinho: An efficient and general plant and animal pre-miRNA predictor for genomic and deep sequencing data

**DOI:** 10.1186/s12859-015-0594-0

**Published:** 2015-05-29

**Authors:** Susan Higashi, Cyril Fournier, Christian Gautier, Christine Gaspin, Marie-France Sagot

**Affiliations:** 1 ERABLE team, Inria Grenoble Rhône-Alpes, Montbonnot Saint-Martin, 38330 France; 2 Université de Lyon, F-69000, Lyon; Université Lyon 1; CNRS, UMR5558, Laboratoire de Biométrie et Biologie Evolutive, Villeurbanne, F-69622 France; 3 INRA, UBIA & Plateforme Bioinformatique, 24 Chemin de Borde Rouge, Auzeville, POBOX 5627, Castanet Tolosan, 31326 France

**Keywords:** microRNA, Prediction, Free-energy, Quadratic algorithm, Whole genome & sRNAseq, Plant/animal

## Abstract

**Background:**

Several methods exist for the prediction of precursor miRNAs (pre-miRNAs) in genomic or sRNA-seq (small RNA sequences) data produced by NGS (Next Generation Sequencing). One key information used for this task is the characteristic hairpin structure adopted by pre-miRNAs, that in general are identified using RNA folders whose complexity is cubic in the size of the input. The vast majority of pre-miRNA predictors then rely on further information learned from previously validated miRNAs from the same or a closely related genome for the final prediction of new miRNAs. With this paper, we wished to address three main issues. The first was methodological and aimed at obtaining a more time-efficient predictor, however without losing in accuracy which represented a second issue. We indeed aimed at better predicting miRNAs at a genome scale, but also from sRNAseq data where in some cases, notably of plants, the current folding methods often infer the wrong structure. The third issue is related to the fact that it is important to rely as little as possible on previously recorded examples of miRNAs. We therefore also sought a method that is less dependent on previous miRNA records.

**Results:**

As concerns the first and second issues, we present a novel alternative to a classical folder based on a thermodynamic Nearest-Neighbour (NN) model for computing the free energy and predicting the classical hairpin structure of a pre-miRNA. We show that the free energies thus computed correlate well with those of RNAfold. This novel method, called Mirinho, has quadratic instead of cubic complexity and is much more efficient also in practice. When applied to sRNAseq data of plants, it gives in general better results than classical folders. On the third issue, we show that Mirinho, which uses as only knowledge the length of the loops and stem-arms and the free energy of the pre-miRNA hairpin, compares well with algorithms that require more information. The results, obtained with different datasets, are indeed similar to those of other approaches with which such a comparison was possible. These needed to be publicly available softwares that could be used on a large input. In some cases, Mirinho is even better in terms of sensitivity or precision.

**Conclusion:**

We provide a simpler and much faster method with very reasonable sensitivity and precision, which can be applied without special adaptation to the prediction of both animal and plant pre-miRNAs, using as input either genomic sequences or sRNA-seq data.

**Electronic supplementary material:**

The online version of this article (doi:10.1186/s12859-015-0594-0) contains supplementary material, which is available to authorized users.

## Background

MicroRNAs (miRNAs) are single strand non-coding RNAs of approximately 22nt which are known to regulate gene expression at the post-transcriptional level. MiRNAs were recognised for controlling, in different organisms, several basic pathways, such as those involved in stress resistance, apoptosis during wing development in *D. melanogaster*, and cell proliferation in the same organism [1-3].

Given the ubiquity of these regulatory molecules and their functional importance, it became crucial to develop methods for the prediction and analysis of miRNAs. As a consequence, in the last few years, a plethora of such software have been developed. More details on the current methods are available in Additional file 1: Table S1 and in Section “Compared methods”. For a review of the existing ones for (pre-)miRNA prediction, see [4,5].

Despite all the effort put in developing such methods, there remains a number of issues that need to be addressed: (i) to predict the characteristic hairpin structure of a pre-miRNA, the vast majority of the existing software rely on a folding algorithm of cubic time complexity which is suitable when the input is small enough, but can become impracticable when the size of the input increases; (ii) for longer pre-miRNAs (such as in plants), such folding methods moreover produce hairpin structures different from the ones provided in MIRBASE [6] which uses sRNAseq data to do so; (iii) together with folding, most methods then rely on further information that must be learned from previously validated miRNAs of closely related genomes (at a minimum within the same clade, plant or animal) for the final prediction of new miRNAs in order either to set the parameters of the model or to restrict the search to a limited space.

**Figure 1:**
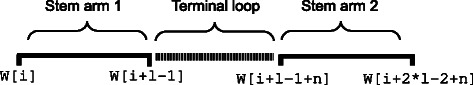
Stem-loop representation and coordinates. The black lines represent the stem-arms and the stripped line represents the terminal loop. *W* is the input sequence of length *N* and *i*∈{0..*N*−2∗*l*−*n*}.

In this paper, we address all three issues. The search for pre-miRNAs is concentrated on regions with the same length as the two stem-arms separated by the length of the terminal loop (see Figure 1). The direct application to *sRNAseq* data guarantees a better quality in the prediction of the pre-miRNA structures. A *quadratic* time complexity algorithm improves the practical efficiency of the free energy computation. As neither of the three attributes used (lengths of the stem-arms and terminal loop, and free energy) are species-specific within the animal or the plant kingdom (they differ only between these two kingdoms), the method can easily be applied for predicting pre-miRNAs in either clade.

Importantly, while the method we provide is thus much simpler, faster, and general to use, we also show on a set of examples that its sensitivity, precision, and specificity are as good as those of other methods, in some cases even better. Moreover, we show that the secondary structures predicted by MIRINHO are much closer to the ones available in MIRBASE than for the other compared methods.

## Methods

### Dataset

To explore a variety of data types from several species, we used six datasets. The sRNAseq data and the genomes were obtained from the NCBI. The annotations concerning the known (pre-)miRNAs were obtained from MIRBASE release 20 [6]. The datasets are organised as follows:

- D1: the chromosomes of six metazoan species:
  - Chromosome 25 from *Bos taurus* (27 miRNAs)
  - Chromosome I from *Caenorhabditis briggsae* (14 miRNAs)
  - Chromosome 2R from *Drosophila simulans* (36 miRNAs)
  - Chromosome 25 from *Gallus gallus* (6 miRNAs)
  - Chromosome 22 from *Gorilla gorilla* (8 miRNAs)
  - Chromosome 19 from *Mus musculus* (60 miRNAs)
- D2: the genomic sequence of length 5,000nt extracted from the human chromosome 1 and taken from the dataset used by [7].
- D3: the artificial dataset compiled by [8]. This dataset is composed of 168 pseudo pre-miRNAs and 163 true human pre-miRNAs. The pseudo pre-miRNAs are sequences that form a hairpin structure; however, they are not functional because they are located in coding sequence regions (CDS). This dataset is available at http://bioinfo.au.tsinghua.edu.cn/mirnasvm/.
- D4: an artificial dataset compiled by us. For each of the three chromosomes listed below:
  - Chromosome III of *Caenorhabditis elegans* (44 miRNAs)
  - Chromosome 2R of *Drosophila melanogaster* (92 miRNAs)
  - Chromosome 19 of *Homo sapiens* (234 miRNAs)
  10 miRNAs were randomly chosen together with 100nt both up and downstream. Each fragment (miRNA + extension) was flanked by sequences of the same length, which were generated based on the nucleotide distribution of the given chromosome. In the end, we obtained three different sequences of ∼ 4,265nt that were given as input to CSHMM, MIReNA, Mirinho, and miRPara (see Section “Compared methods”).
- D5: genomic data of three insects that are of special interest for our group:
  - *Acyrthosiphon pisum* genome assembly version 2 (123 miRNAs)
  - *Culex quinquefasciatus* genome assembly version 1 (120 miRNAs)
  - *Heliconius melpomene* genome assembly version 1.1 (101 miRNAs)
- D6: genomic and sRNAseq data of plants. We used the sequence of chromosome 4 of *Arabidopsis thaliana* (version 2.0) and the whole genome of *Arabidopsis lyrata* (version 1.0), as well as the high-throughput small RNA sequencing data from the same species with, respectively, NCBI GEO accession number GPL3968 and GSE18077/GSE20442. The chromosome 4 from *A. thaliana* and the genome of *A. lyrata* contain, respectively, 57 miRNAs and 384 miRNAs.

### Pre-processing input sequences with small RNA sequencing data

When sRNAseq data are available, they may be used to pre-process the input sequence, focusing the search only on transcribed regions. We implemented this functionality in a separate module, called PROCIN, that is optionally performed before the core algorithm for the prediction of pre-miRNAs.

First, the small RNA reads are mapped against the genome using BOWTIE [9]. The genome sequence and the respective mapping file (extension.sam) produced by BOWTIE are then given to PROCIN. Using the SAM file, the regions with at least one read are extracted from the genome sequence. If the region is smaller than 70nt (approximate length of a pre-miRNA), we extend it by 70nt up and downstream to guarantee that the whole pre-miRNA sequence will be covered. Both values – number of reads covering the region and length of this region – may be given as parameters to this module.

**Figure 2:**
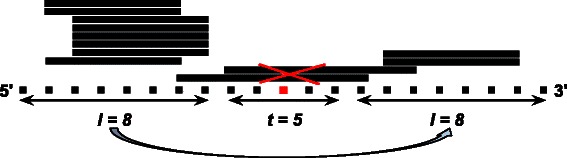
Using sRNAseq data to set the parameters of MIRINHO. The solid lines are the reads that align to the pre-miRNA sequence represented by the dotted line (the dots are the coordinates of the pre-miRNA sequence). The point in red is the middle coordinate of the pre-miRNA that serves to eliminate reads that align to the terminal loop and may mislead the procedure of determining the parameters. *l* is the length of the stem-arm and *t* is the maximum length of the terminal loop.

For each (extended) pre-miRNA sequence, the lengths of the stem-arm and of the terminal loop are set as follows (see Figure 2 for an example). The highest read stack is identified, either on the 5’ or on the 3’ arm. If this stack is on the 5’-arm, the end position *i* of the right-most aligned read is considered and the length of the stem-arm is set to *l*=*i*+1. If the stack is on the 3’-arm, *p* is the length of the pre-miRNA, and *j* is the start position of the left-most aligned read, the length of the stem-arm is set to *l*=*p*−*j*+1. The stem-arm is then “mirrored” to the opposite stem-arm and the distance between the two arms is the maximum length of the terminal loop. One should notice that reads aligning to the terminal loop could mislead this procedure. We solved this problem when establishing the lengths by disregarding all the reads that aligned to the middle coordinate of the putative pre-miRNA, in an attempt to focus only on the reads aligning to the arms.

One should notice that other molecules, such as rRNAs and tRNAs that may have a read stack similar to the one of a miRNA, can be recovered by MIRINHO if they fold themselves into stable stem-loops. Since rRNAs and tRNAs are molecules that are usually well annotated, a simple solution is to mask these regions before running MIRINHO, for example with MASKFASTA from the BEDTOOLS package [10].

### Screening the genome to identify potential pre-miRNAs

The algorithm of MIRINHO begins with a pre-treatment of the data in order to identify the input for the alignment step. This is done without loss of information at this step, meaning that all the positions in the genome are verified to determine whether they may contain a pre-miRNA. This is done by screening the genome as follows.

As one may see in Figure 1, we set a sliding window of length *l*=25, which represents the length of the stem. For each stem-arm represented by *s* *t*_1_=[*w*_*i*_,...,*w*_*i*+*l*−1_], we look for its putative stem-arm pair *s* *t*_2_=[*w*_*i*+*l*+*n*−1_,...,*w*_*i*+2∗*l*+*n*−1_]*n* nucleotides away from the first one, with *n* between 5 and 20. In order to set the length of the stem, we consider the mean length of a miRNA (i.e., 22nt), and to ensure that the whole miRNA is considered in the alignment, an offset of 3nt, equivalent to the standard deviation (i.e., *σ*=3.33) of the lengths of the miRNAs in MIRBASE (release 20), was added. Using this strategy, we guarantee that all the miRNAs that are smaller than 25nt will be considered. The lengths of the terminal loop are set on the basis of the distribution of the loop lengths for metazoan pre-miRNAs (MIRBASE release 20) as one may see in Additional file 1: Figure S11. Each pair (*l*, *n*) will be an input for the alignment algorithm.

### Assessing the potential pre-miRNAs

Each pair of putative stem-arms screened in the step described in the previous section is given to an alignment algorithm in order to evaluate whether it can form a stable stem-loop structure. For that, we implemented the Needleman & Wunsch global alignment algorithm [11] with a scoring strategy based on the Nearest-Neighbour energy model. Instead of using the sum of the integer penalties for gaps, matches and mismatches, the alignment is assessed according to the free energy of each two consecutive nucleotides in the alignment. This is explained in more detail next.

### Nearest-neighbour model

To model the free energy change for the folding of these RNAs, one can use the thermodynamic Nearest-Neighbour (NN) energy associated to each type of motif in the structure. By summing up the energy increment of each motif, it is possible to obtain a reasonable approximation of the free energy change for folding an RNA or, in other words, to obtain a measure of the stability of an RNA molecule [12,13].

The motifs forming an RNA structure are determined by the base-pairs AU, GC and GU. The arrangement of these base pairs can shape into the different types of motifs, such as helices, bulge loops, and internal loops. The stabilising motifs are: the Watson-Crick helix represented by the stacking of at least two base pairs; and a dangling end which is a single base at the end of a helix. The destabilising motifs are of three types: the hairpin loop which is composed of non-canonical base pairs closed by one canonical base pair; the bulge loop which is an arrangement of unpaired nucleotides in one of the strands of a helix; and finally, the internal loop which includes unpaired nucleotides in both strands of a helix. There exist three more types of destabilising motifs: exterior loop, pseudoknot, and multibranch. The first two are not present in a pre-miRNA stem-loop structure and will therefore not be explored in detail here [13,14]. The third one (multibranch loops) may occur in the terminal loop of longer hairpin sequences (such as plant pre-miRNAs). Pre-miRNAs with such a kind of motif will be recognised if the hairpin is stable enough. However, it will not be considered in the computation of the free energy because our method only uses the stem-arms to calculate such energy and the terminal loop to separate these two arms.

As mentioned before, to compute the free energy of an RNA structure, it is necessary to sum the increments according to the type of the motif. The equations presented hereafter describe how to compute the free energy associated to each such type.

The energy of a dangling end depends only on the base-pair before the dangling nucleotide and on this latter. For all the other types of motifs, the equations are given below. The energy of an internal loop is computed by means of Equation 1:

(1) $$\begin{array}{@{}rcl@{}} \Delta G_{Internal} &=& \Delta G_{i}(n) + (\Delta G_{a} * | n_{1} - n_{2}|) + \Delta G_{m1} \\ &&+\Delta G_{m2} + (\Delta G_{ru} * \lambda)  \end{array} $$

where *Δ* *G*_*i*_(*n*) is the initiation energy to form an internal loop of *n*≤30 unpaired nucleotides; *Δ* *G*_*a*_=0.6 is the asymmetry penalty multiplied by the absolute value of the difference between the number of unpaired nucleotides in each strand; *Δ* *G*_*m*1_ and *Δ* *G*_*m*2_ are the energy of the first and the last mismatches in the internal loop; and *Δ* *G*_*ru*_=0.7 is the penalty for an RU closure, where *R*={*A*,*G*} and *λ* is the lambda function which returns 0 or 1, corresponding, respectively, to the presence or absence of, in this case, the RU closure.

For the bulge loops, one should use Equation 2:

(2) $$ \begin{aligned} \Delta G_{Bulge (n=1)} =&\; \Delta G_{i}(1) + \Delta G_{C} + \Delta G_{s} - RT ln(t)\\ &+ (\Delta G_{ru} * \lambda)\Delta G_{Bulge (n>1)} = \Delta G_{i}(n) \end{aligned}  $$

where *Δ* *G*_*i*_(*n*) is the energy required to form a bulge with *n*≤30 unpaired nucleotides; if the bulge is comprised of the nucleotide C only, and there is at least one more C not in the bulge (meaning, it is paired with a G), one should add the C bulge penalty *Δ* *G*_*C*_=−0.9 kcal/mol; *Δ* *G*_*s*_ is the base pair stacking around the bulge; *t* is the number of possible loop conformations with identical sequence; *R*=8.3144621 J/mol K is the gas constant and *T*=310.15 K is the temperature in kelvin. Notice that for bulges and helices, *Δ* *G*_*ru*_=0.45 and is referred to as the penalty for an RU end (and not closure as for internal loops).

For bulges and internal loops larger than 30 nucleotides (*n*>30), Equation 3 should be applied instead:

(3) $$\begin{array}{@{}rcl@{}} \Delta G_{n > 30} = \Delta G_{i}(30) + 1.75 \times RT \times ln(n/30)  \end{array} $$

Finally, for a helix, one should apply Equation 4:

(4) $$\begin{array}{@{}rcl@{}} \Delta G_{helix} = \sum \Delta G_{stck} + \Delta G_{sym} + (\Delta G_{ru} * \lambda)  \end{array} $$

where *Δ* *G*_*stck*_ is the stacking energy of each two consecutive base pairs; *Δ* *G*_*sym*_ is the symmetry correction for self complementary duplexes; and *Δ* *G*_*ru*_=0.45 is, as mentioned before, the RU end penalty.

All the thermodynamic NN energies used in this work, as well as the equations described above, were obtained in the NEAREST NEIGHBOR DATABASE (NNDB) [14,15].

### Algorithm

We implemented a global alignment algorithm (see the Section “Needleman-Wunsch algorithm” below) and an alignment assessment approach based on the NN energy model to measure the stability of pre-miRNA candidates, that is explained below.

We define the alphabet *Σ*={*M*_*xy*_,*S*_*xy*_,*I*_*xy*_,*D*_*xy*_}, where the symbols correspond, respectively, to *Match*, *Mismatch*, *Insertion* and *Deletion*, and *x*,*y*∈{*A*,*U*,*C*,*G*,−}. The definition of an alignment of two putative stem-arms, *s* *t*_1_ and *s* *t*_2_, is a vector comprised by the symbols in *Σ*, such that *a* *l* *i* *g* *n*(*s* *t*_1_,*s* *t*_2_)=*v* and *v*= [*v*_*i*_,*v*_*i*+1_,...,*v*_*n*_], where *v*_*i*_∈*Σ*.

To determine the stability of a pre-miRNA stem-loop, we go through vector *v* and sum up the free energy of each pair (*v*_*i*_,*v*_*i*+1_) according to the type *t* of the motif in which it is inserted. For that, we use Equation 5 below to compute the energy of each motif in the structure:

(5) $$\begin{array}{@{}rcl@{}} \epsilon(t) = k(t, n) + \sum_{i}^{n-1} e(v_{i}, v_{i+1})  \end{array} $$

where *t* is the motif type that can be an internal loop, a bulge loop or a helix. The value *k*(*t*,*n*) accounts for penalties associated to the motif *t*, which appears *n* times in the structure. For example, for a motif of type *t*=*h* *e* *l* *i* *x*, one should consider the symmetry correction for self-complementary duplexes *Δ* *G*_*sym*_ (see Equation 4). Finally, the function *e* returns the energy associated to the pair (*v*_*i*_,*v*_*i*+1_).

We then sum all the energies related to the different types of motifs to obtain the total free energy *E* of the structure using Equation 6:

(6) $$\begin{array}{@{}rcl@{}} E = \sum \epsilon(t)  \end{array} $$

where *t* is again the different types of motifs a given structure can have.

#### Needleman-Wunsch algorithm

Since the aligned sequences are similar in length, we implemented the DP global alignment algorithm described by Needleman and Wunsch, which will try to align every nucleotide in the sequences. The recurrence for this algorithm is presented in Equation 7:

(7) $$\begin{array}{@{}rcl@{}} W(i,j) = max \left\{ \begin{array}{l l} W(i-1, j-1) + f(s_{i}, s_{j}) \\ W(i, j-1) + \gamma\\ W(i-1, j) + \gamma \end{array} \right.  \end{array} $$

where *f*(*s*_*i*_,*s*_*j*_) is the function returning the score or penalty for, respectively, a match or a mismatch, and *γ* is the penalty for a gap. Using this recurrence one should take, in the worst case, $\mathcal {O}(n^{2})$ time to align two sequences of length *n* [11].

Considering that a stable hairpin structure should not contain very large bulges nor internal loops, an ideal alignment should be concentrated around the main diagonal of the dynamic programming (DP) matrix. Instead of using the whole matrix, the user can therefore constrain the alignment to this diagonal and prune parts of the bottom-left and top-right corners of the matrix, thus saving time in the computation of the free energies with a small loss.

A parameter *dw* (diagonal width) is established that depends on the length of the aligned sequences and on a compromise between sensitivity and precision in relation to the version that uses the full matrix (see the Section “Computing time” to determine how to set an appropriate value for this parameter). This parameter is associated to the number of consecutive gaps (i.e., the length of the bulges) the alignment may have.

#### RNA secondary structure prediction algorithm

To facilitate the comparison of the complexity of each algorithm, we present here a description of the method for predicting an RNA secondary structure. The first such method is the one that was described by Nussinov [16]. The algorithm proposes a maximisation of the number of base pairs to find the best structure. For each position *i* in the sequence, one should verify all the possible cases: (a) *i*,*j* base pair; (b) *i* is unpaired; (c) *j* is unpaired; (d) *i*,*j* base pair with, respectively, *k* and *k*+1. The recurrence for this algorithm is presented in Equation 8 [17]:

(8) $$\begin{array}{@{}rcl@{}} E(i,j) = max \left\{ \begin{array}{l l} E(i+1, j-1) \text{if i and j base pair} \\ E(i+1, j) \\ E(i, j-1) \\ {max}_{i < k <j} [ E(i,k) + E(k+1,j) ]\\ \end{array} \right.  \end{array} $$

Clearly, filling each cell in the DP matrix takes $\mathcal {O}(n)$ time, and since there are $\mathcal {O}(n^{2})$ cells, the complexity for the whole procedure is in $\mathcal {O}(n^{3})$. However, maximising the number of base pairs is a naïve approach; a more realistic one is to minimise the free energy of the structure, for example as proposed by Mathews [12]. The recurrence for the latter algorithm is presented in Equation 9:

(9) $$\begin{array}{@{}rcl@{}} E(i,j) = min \left\{ \begin{array}{l l} E(i+1, j)\\ E(i, j-1)\\ {min}_{i < k <j} [ E(i,k) + E(k+1,j) ]\\ P(i,j) \text{if i and j base pair} \end{array} \right.  \end{array} $$

To minimise the free energy, one more table *P* is required to store the different types of motifs a structure can have, although the complexity in the worst case remains the same, namely in $\mathcal {O}(n^{3})$ [12,13].

### Setting the energy threshold

To determine an appropriate energy threshold for the prediction of pre-miRNAs, three approaches were tested. The first two were ROC (Receiver Operating Characteristic) curves, one using the insect genomes from dataset D5 (Additional file 1: Figure S3) and the other the pre-miRNA dataset D3 (see Additional file 1: Figure S4). In the third approach, we used a set of random genomes generated according to a multinomial distribution and compared the pre-miRNAs predicted in such genomes against those predicted in the original metazoan genomes from dataset D1. The reasoning behind this strategy is that, if the energy model is robust enough, there should exist an energy threshold that is able to differentiate the stable hairpin structures from the randomly generated ones in which the base pairs would be established by chance.

**Figure 3:**
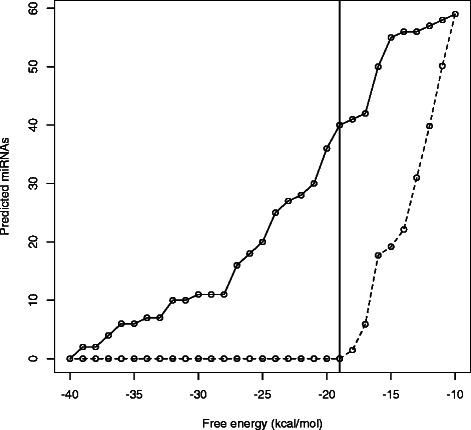
Setting energy threshold. Number of TP pre-miRNAs predicted when using the original and the random genomes of *M. musculus*. The vertical line represents the energy (−19 kcal/mol) that better distinguishes true from false pre-miRNAs.

The nucleotide frequency distribution in the original metazoan genomes was used to generate the respective random genomes. After that, the prediction of the pre-miRNAs was performed on both versions (original and random) of each genome. We then chose as threshold the biggest energy for which the number of true miRNAs remains zero in the random genome, as can be seen in Figure 3 and Additional file 1: Figures S5, S6, S7, S8 and S9. To define a “true” miRNA in the random genome, we considered only if its coordinates were the same as those of a true miRNA in the original genome from which the random one was created. Using this approach, the selected genomes had the thresholds presented in Table 1.

**Table 1:** **Computing time comparison**

**Method**	**Time**
Mirinho	0.998
miRPara	68.008
MIReNA	989.958
CSHMM	1824.474

Computing time (in seconds) for the prediction of pre-miRNAs in a sequence of length 5,000nt (dataset D2), on a Mac OS X 10.6.8, 2.7GHz Intel Core i7.

In the first approach, the best operating point was providing an acceptable sensitivity and a higher specificity, which was being overestimated due to the inability to define a true negative pre-miRNA at a genomic scale (see Section “D5: insect genomes”). Moreover, the threshold (*e*=−16.3) established with this approach was leading to a very weak precision. We then proceeded to use the second and third approaches which produced more reasonable results, meaning having good sensitivity and precision. The thresholds set with the latter approaches are very similar, *e*=−21 kcal/mol for the second and *e*=−20.6 kcal/mol for the third. The default energy threshold is set to −20.6 kcal/mol, that is the mean of the energies mentioned in Table 1.

Once the secondary structure is computed by MIRINHO, we may verify if the reads aligned on the locus mimic the constraints consistent with Dicer processing by using an *a posteriori* script called CHECKDICER. We check the position of the reads within the hairpin (on the mature, star or terminal loop), allowing facultative read overlaps between these three regions. Such overlaps may cause false positives. At the same time, directly discarding the candidates with overlapping reads may also result in false negatives. To thus allow a flexible tradeoff between FP and FN, the user may provide two parameters to CHECKDICER: (i) the percentage of the overlap for each read; and (ii) the percentage of the number of reads overlapping. The user may thus choose to be strict with Dicer processing by setting the first parameter to zero (risk of FN); or to allow small percentages for one or both values (risk of FP). It is important to specify that the Dicer processing validity check implemented in CHECKDICER has not been used in any of the analyses that were performed.

### Comparison with other methods

#### Compared methods

To compare the accuracy of our method with the one of other predictors, we first made an extensive search of the available ones (see Additional file 1: Table S1). We put aside the predictors that required other kinds of input files than just the fasta sequence, as well as those incompatible with the Unix system. Web-servers were also disregarded because there always is a restriction to the length of the sequence that may be input. The methods that remained were CSHMM, MIRENA, and MIRPARA. Notice that as one of our main contributions is the efficiency in the prediction of pre-miRNAs in relation to other methods that use cubic complexity algorithms, it was natural to compare MIRINHO to methods that adopt this kind of algorithm. However, we also included in the comparison a method such as CSHMM which does not use a cubic algorithm for the prediction of miRNAs.

Since the set of input parameters differs for each method, it is not a trivial task to set them in accordance with the data and at the same time be fair in the comparison. We thus decided to apply the methods with default parameters. However, we adapted one aspect that was common to all the methods: the set of known (pre-)miRNAs. All the methods were trained, when required, with animal (pre-)miRNA sequences. A description of each method, and how each was set up is given below.

CSHMM uses a Context-Sensitive Hidden Markov Model to predict pre-miRNAs [7]. To set the initial parameters for CSHMM, we used the secondary structures of the kingdom metazoan that are available in MIRBASE release 20. To generate the likelihood score, the metazoan hairpin sequences were used as positive training set, and as negative instances the sequences employed by the authors.

MIRENA applies a series of consecutive filters to determine a pre-miRNA and does not require a training step [18]. It provides different starting points for the prediction depending on the type of file given as input. We set the parameter to allow for a genomic input (-M option). The set of known mature miRNAs required was from the same metazoan kingdom, taken from MIRBASE release 20.

MIRPARA (version 6.2) is an SVM implementation trained with sequences from MIRBASE [19]. It makes available a script to generate the model according to the MIRBASE release and to the desired organism(s)/clade. In our case, we chose the model trained with the metazoan pre-miRNAs of MIRBASE release 20. It is worth observing that among the compared methods, MIRPARA is the only one that also predicts the position of the miRNA within the pre-miRNA.

To compare the performance of our method when using sRNAseq data, we used MIRDEEP which is a method for the discovery of miRNAs from deep sequencing data [20]. To obtain potential pre-miRNAs, the authors use information of the mapped reads against the genome. Pre-miRNAs with an unlikely structure are discarded and the core algorithm computes a probabilistic score related to the structure and to the signature of the pre-miRNA candidate. It is worth reminding that the core algorithm of MIRINHO only requires a fasta sequence; sRNAseq data, when available, are used to pre-process the input sequence by considering only the mapped regions, as is described in Section “Pre-processing input sequences with small RNA sequencing data”.

To analyse the quality of the predicted structures, we used RNAFOLD [21] and MIRNAFOLD [22]. The first is a classical method for predicting an RNA secondary structure through energy minimisation. The second is a method for predicting a hairpin structure that takes into account specific criteria (such as the length of the stem, the percentage of nucleotides, the size of the terminal loops) related to known hairpins from MIRBASE, and verifies if these are present in the query structure. MIRNAFOLD is moreover, as far as we know, the only other method that has quadratic complexity for predicting a pre-miRNA structure. For more details on how each of the methods were used, see the next section.

#### Pre-miRNA hairpin structure in plants

To evaluate the performance of each method in the prediction of pre-miRNAs, we used as measures sensitivity, precision and specificity. The first is the proportion of true pre-miRNAs that are correctly predicted, the second is the fraction of predicted pre-miRNA candidates that are real pre-miRNAs, and the third is the proportion of false pre-miRNAs that are correctly excluded:

(10) $$\begin{array}{@{}rcl@{}} Sensitivity = \frac{TP}{TP+FN}  \end{array} $$

(11) $$\begin{array}{@{}rcl@{}} Precision = \frac{TP}{TP+FP}  \end{array} $$

(12) $$\begin{array}{@{}rcl@{}} Specificity = \frac{TN}{TN+FP}  \end{array} $$

where TP stands for True Positive, TN for True Negative, FP for False Positive, and FN for False Negative.

To compute the number of true pre-miRNAs predicted by each method, we did the following. For a given species, there is a control set *C*={*c*_*j*_,*c*_*j*+1_,...} of miRNAs that are considered to be true miRNAs according to MIRBASE, where *j*∈{1..*n*} and *n* is the number of true miRNAs for a given species. For each predicted pre-miRNA, denoted by *ppm*, we verified whether one of its arms fully covers a control miRNA, denoted by *c* *m*_*j*_. If that was the case, we accounted for one TP. If the same *ppm* covered more than one control miRNA, we considered just the one with the best prediction score according to each method. It is worth observing that a large proportion of the miRNA sequences in MIRBASE are not supported by experimental evidence. However, it is considered as a reference database.

## Results and discussion

### Regression analysis of the free energies

To verify how close we get to the algorithms based on a secondary structure prediction, we present a regression analysis between the energies of the pre-miRNAs corresponding to the true positive pre-miRNAs predicted by MIRINHO and their energies when predicted by RNAFOLD [23].

**Figure 4:**
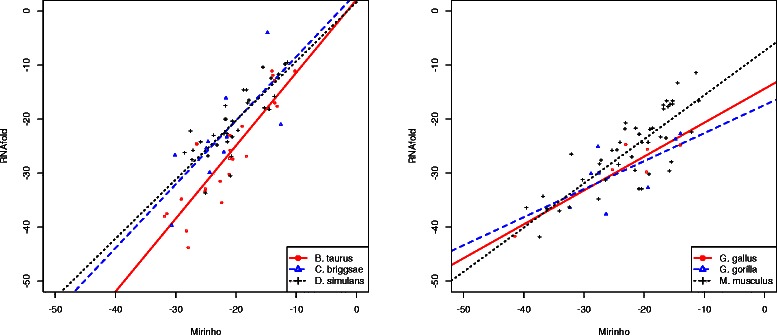
Regression analysis. Regression analysis of the energies predicted by MIRINHO and RNAFOLD. On the left, chromosome 25 of *B. taurus*, chromosome I of *C. briggsae*, chromosome 2R of *D. simulans*. On the right, chromosome 25 of *G. gallus*, chromosome 22 of *G. gorilla*, chromosome 19 of *M. musculus*.

Figure 4 shows the relationship of the energies for the true positive pre-miRNAs of the chromosomes in dataset D1. We consider as the dependent variable the energies of MIRINHO and as the independent variable the energies of RNAFOLD. As we can see, the energies are quite close to each other with, in general, bigger energies predicted by MIRINHO. This provides reasonable evidence that our method approximates well the free energy of hairpins.

### Computing time

As mentioned (in Section “Algorithm”), we further improved the alignment algorithm by pruning the DP matrix and focusing on its diagonal only.

To establish the size of the diagonal portion of the DP matrix that should be explored, we assessed different values for the parameter *dw* (diagonal width). Additional file 1: Table S2 shows the results of the evaluation performed to determine a reasonable value for this parameter. The values for *dw* were evaluated empirically; they varied from 4 to 6. A too small value for *dw* would constrain the alignment to a very limited space around the diagonal part of the DP matrix, that is, would permit a few or almost no bulges or internal loops. This situation would not represent the real structure of a stem-loop and that is why we chose as minimum value *d* *w*=4. On the other hand, a very large value for *dw* would not achieve the goal of the pruning strategy, that is time efficiency. In our experiments, the best results were obtained when using *d* *w*=4 or *d* *w*=5, which corresponds to the maximum number of unpaired nucleotides in the stem formed by both strands.

The user of MIRINHO is given the freedom to compute the whole matrix instead of only its diagonal for a given value of *dw*. In this case, *dw* should be set equal to the length of the stem-arm (option -a).

Using this pruning strategy, the region explored by the alignments is much smaller and the method performs, in general, 30*%* faster than the original version. Sensitivity and precision remain similar between the original and the optimised versions; in the great majority of the cases it remained the same.

Time efficiency is even more evident when comparing our method to other predictors, such as CSHMM, MIRENA, and MIRPARA. Table 2 presents the computing times for the prediction of pre-miRNAs in a sequence of dataset D2, running under a Mac OS X 10.6.8, 2.7GHz Intel. As one can see, our method is indeed much faster than the others, making the prediction of pre-miRNAs much more feasible.

**Table 2:** **Setting the energy threshold**

**Species**	**Energy threshold**	**Chromosome**	**GC%**
*C. briggsae*	−16	I	37,76
*M. musculus*	−19	19	42,73
*G. gorilla*	−19	22	47,74
*D. simulans*	−21	2R	43,93
*G. gallus*	−24	25	54,96
*B. taurus*	−25	25	46,96
*C. elegans*	−	III	35,75
*D. melanogaster*	−	2R	41,84
*H. sapiens*	−	19	50,06

Energy thresholds, obtained with the methodology mentioned in Section “Setting the energy threshold”, and the GC% of the different chromosomes from dataset D1, including the ones for test from dataset D4 (three last lines). To choose the genomes, we mainly considered the GC content (varying from 37% to 54%), as this plays an important role in determining a hairpin structure.

To show this, we compared the prediction time of MIRINHO, CSHMM, and MIRPARA. To facilitate the comparison of the predicted pre-miRNAs, we used dataset D4, as did the authors of CSHMM. All three software were submitted to a cluster queue of 29 hours. MIRINHO finished its job after 5 hours, while the other two exceeded the 29 hours without finishing their prediction, with no reported result.

### Pre-miRNA hairpin structures

Information on the length of the stem-arms and terminal loop may produce higher quality pre-miRNA structures. When the search is made at a genomic scale, such precise information is not available. However with sRNAseq data, the length of the stem-arms and terminal loop may be naturally inferred from the alignment of the reads against the genome (see Section “Pre-processing input sequences with small RNA sequencing data”). The way we screen the input sequence thus allows for a direct application of MIRINHO to sRNAseq data, thus enriching the prediction and the quality of the hairpin structures.

To demonstrate this, we first set the parameters *l* (length of the stem-arm) and *n* (length of the terminal loop) using dataset D6, as described in Section “Pre-processing input sequences with small RNA sequencing data”. We then gave the pre-miRNA sequence and the latter parameters as input to MIRINHO, RNAFOLD, and MIRNAFOLD. For MIRINHO, we set the stem-arm length to *l* using option -a and the minimum and maximum length of the terminal loop to *n* using options -n and -x respectively. Given that RNAFOLD is a method for predicting the secondary structure of an RNA in general, we used the option -C to force the structure to be a hairpin. We then required that the stem-arm regions, each of length *l*, were paired, and that the terminal loop region of length *n* was unpaired. For MIRNAFOLD, we established as the sliding window parameter the length of the whole pre-miRNA, that is, *l*+*n*+*l*.

It is worth observing that the structures in MIRBASE are also predictions. We however use them as a standard because MIRBASE is considered as a reference database: it is being constantly updated leading to new releases and it incorporates criteria based on experimental (i.e., sRNAseq) approaches to reinforce the evidence provided [24]. In order to compare the structures predicted by the three methods, we considered four criteria: (i) the number of internal loops within the stem; (ii) the number of bulges within the stem; (iii) the length of the predicted stem-arm; (iv) the length of the predicted terminal loop. For each predicted structure, we verified which method produced the best result. This corresponded to the predicted structure that produced values that are closest to those of the structure in MIRBASE. For example, if the MIRBASE structure *s* has 3 bulges, and RNAFOLD predicted a structure with 2 bulges while MIRINHO predicted one with 1 bulge, the first method would be considered the best one.

From the set of 57 pre-miRNAs of chromosome 4 of *A. thaliana*, we randomly chose 10 sequences for the prediction of the hairpin structures by the three methods. In the end, MIRINHO obtained the closest structure in 80% of the cases, RNAFOLD was the second with 50%, and MIRNAFOLD the third with 40% (the predicted structures are present in the Additional file 2 for MIRBASE, MIRINHO, and MIRNAFOLD, the ones for RNAFOLD are present in the Additional file 3).

**Figure 5:**
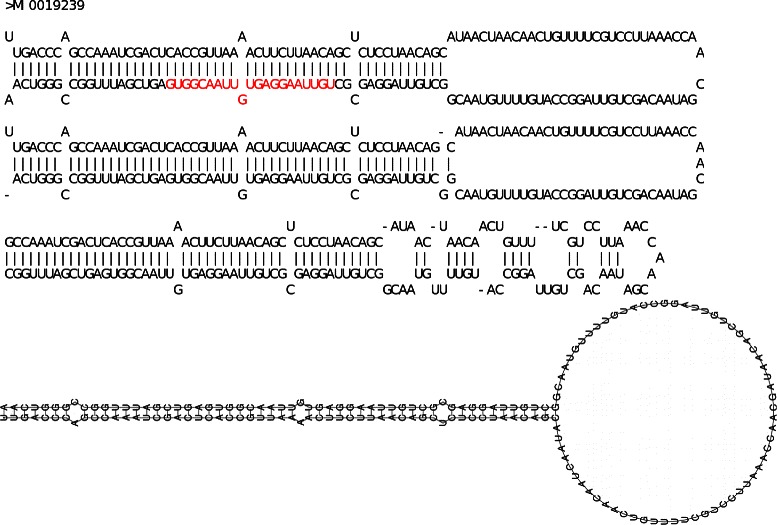
Predicted secondary structures (RNAFOLD). From top to bottom: gold standard structure in MIRBASE (with miRNA coloured in red), and structures predicted by, respectively, MIRINHO, MIRNAFOLD, and RNAFOLD. Secondary structure of pre-miRNA MI0019239, the best prediction was by RNAFOLD with the closest values of stem length, terminal loop length, and number of bulges and internal loops as in MIRBASE.

**Figure 6:**
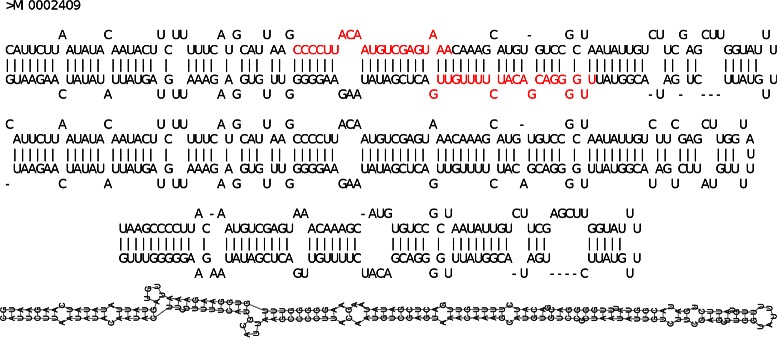
Predicted secondary structures (MIRINHO). From top to bottom: gold standard structure in MIRBASE (with miRNA coloured in red), and structures predicted by, respectively, MIRINHO, MIRNAFOLD, and RNAFOLD. Secondary structure of pre-miRNA MI0002409: the best prediction was by MIRINHO with the closest values of stem length, and number of bulges and internal loops as in MIRBASE.

**Figure 7:**
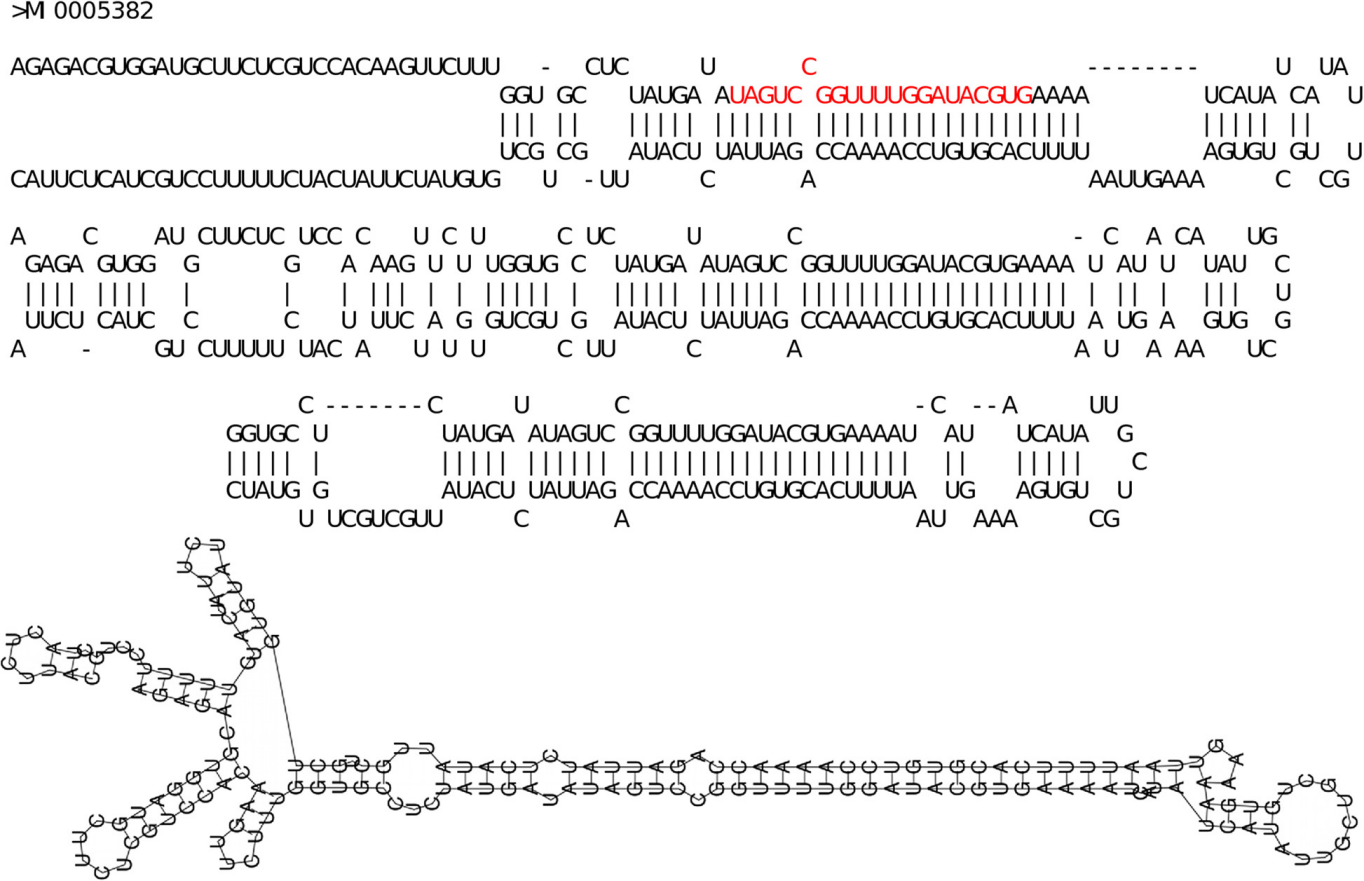
Predicted secondary structures (MIRNAFOLD). From top to bottom: gold standard structure in MIRBASE (with miRNA coloured in red), and structures predicted by, respectively, MIRINHO, MIRNAFOLD, and RNAFOLD. Secondary structure of pre-miRNA MI0005382: the best prediction was by MIRNAFOLD with the closest values of terminal loop length, and number of bulges and internal loops as in MIRBASE.

Figures 5, 6 and 7 show, respectively, cases in which the closest structure was found by RNAFOLD, MIRINHO, or MIRNAFOLD. As we can see, even in the cases where MIRINHO was not the best, it was very close to the best.

To extend the comparison to broader cases, we compared, for the whole set of pre-miRNAs of the *Arabidopsis* genus, the location of the miRNA within the pre-miRNA structure. More specifically, the secondary structures of the pre-miRNAs of *A. thaliana* and *A. lyrata* were computed with MIRINHO and RNAFOLD (using the -C option to force a hairpin structure). The respective miRNAs were then aligned to these structures. RNAFOLD misplaced the miRNAs (putting them in a place different from a stem-arm) 10% of the times, while MIRINHO correctly placed the miRNAs in all the cases.

**Figure 8:**
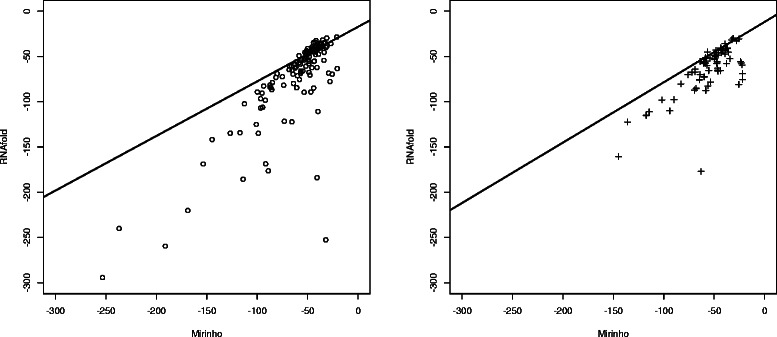
MIRINHO and RNAFOLD energy correlation. Correlation of the free energies predicted by MIRINHO and RNAFOLD for *A. thaliana* (*ρ*=0.80) on the left, and *A. lyrata* (*ρ*=0.73) on the right.

To verify how the energies of MIRINHO and RNAFOLD correlated, we computed a linear regression between the energies predicted by the two methods, as shown in Figure 8. The free energies predicted by both methods are in general similar (*ρ*=0.80 for *A. thaliana* and *ρ*=0.73 for *A. lyrata*), with the energies of MIRINHO being slightly bigger in general.

### Comparison with other methods

To compare the performance of the methods, we used datasets D3, D4, D5, and D6. Specificity was computed only in the case that a true negative pre-miRNA was precisely defined: this happens for dataset D3 in which a true negative is represented by a pseudo pre-miRNA. At a genomic scale, it is not an easy task to define a true negative pre-miRNA; specificity values are thus often overestimated. For this reason, this measure was not included in the genomic scale experiments. It was not included either in the case of sRNAseq because other kinds of transcripts may be expressed, and one may not know a priori if they correspond to a true or to a false pre-miRNA.

#### D3: artificial pre-miRNA dataset compiled by Xue et al.

Table 3 presents a comparison between the different methods when using as input the pre-miRNA dataset D3 compiled by [8]. As one may observe, when considering the measures separately, CSHMM and MIRINHO obtain better sensitivity while for the other measures the overall performance of MIRPARA and MIRENA is better. These results reflect the underlying algorithms used by each of the methods. While CSHMM and MIRINHO are specialised in finding stable hairpin structures, MIRPARA and MIRENA use other characteristics during the prediction. However, at a genomic scale such characteristics do not enable to outperform the two other methods, as one may see in Table 4.

**Table 3:** **Comparison of the sensitivity, precision and specificity using as input the pre-miRNA dataset D3**

	**CSHMM**	**MIRPARA**	**MIRINHO**	**MIRENA**	
	**24760.118s**	**4376.739s**	**1.251s**	**3345.317s**	
Sensitivity	100	88	97	95	
Precision	49	84	72	94	
Specificity	0	84	64	94	
Sensitivity	100	93	98	97	
Precision	38	78	71	91	

The first three lines contain the results when using the original dataset with 163 positive pre-miRNAs and 168 pseudo pre-miRNAs. The last two lines contain the results when using only the 102 positive pre-miRNAs, from the same dataset, annotated with high confidence according to MIRBASE. The energy threshold used in MIRINHO was -20.6 kcal/mol. Values for the three measures are given in percentage.

**Table 4:** **Comparison of the sensitivity and precision using as input the insect genomes of dataset D5**

**Organism**	**Method**	**Sensitivity**	**Precision**
*A. pisum*	Mirinho	*69.92*	0.52
	CSHMM	23.58	0.05
	miRPara	36.59	0.14
	MIReNA	24.39	*3.42*
*C. quinquefasciatus*	Mirinho	*69.17*	0.25
	CSHMM	48.51	0.10
	miRPara	28.33	0.07
	MIReNA	18.33	*2.00*
*H. melpomene*	Mirinho	*78.22*	0.94
	CSHMM	48.51	0.10
	miRPara	58.42	0.23
	MIReNA	31.68	*7.88*

The energy threshold used in MIRINHO was *e* = -20.6. Values are given in percentage.

Considering a practical aspect involving the tradeoff between time and accuracy, one could boost the prediction results in a feasible time by combining MIRINHO and one of the methods that uses specific pre-miRNA characteristics (namely MIRPARA or MIRENA). MIRINHO would thus enable in a first step to quickly obtain all the stable structures, which would then in a second step be given as input to the other methods.

#### D4: artificial pre-miRNA dataset compiled by us

Table 5 presents a comparison between the different methods using as input the pre-miRNA dataset D4. As we can see, in humans, MIRINHO obtains the best sensitivity (70%) and precision (50%) together with CSHMM. As concerns *D. melanogaster*, MIRINHO also has the best sensitivity (80%), while MIRENA gets the best precision (75%). For *C. elegans*, CSHMM obtains the best sensitivity (70%), and MIRENA the best precision (44.44%). This kind of analysis is important to verify how the methods behave when “noise” is flanking the given pre-miRNA, such as in a genomic scale where a lot of information may be confused with the pre-miRNAs.

**Table 5:** **Comparison of the sensitivity, precision, and computing time using as input the pre-miRNA dataset D4**

	**CSHMM**		**MIReNA**		**miRPara**		**Mirinho**	
	**32202.854s**		**918.588s**		**110.261s**		**1.667s**	
	**Precision**	**Sensitivity**	**Precision**	**Sensitivity**	**Precision**	**Sensitivity**	**Precision**	**Sensitivity**
*H. sapiens*	23.08	60.00	*50.00*	10.00	13.00	60.00	*50.00*	*70.00*
*D. melanogaster*	26.92	70.00	*75.00*	30.00	08.00	60.00	61.54	*80.00*
*C. elegans*	29.17	*70.00*	*44.44*	40.00	04.00	20.00	35.71	50.00

The energy threshold used in MIRINHO was *e* = -20.6. The values for sensitivity and precision are given in percentage.

#### D5: insect genomes

To analyse the sensitivity and precision at a genomic scale (dataset D5), we used the genomes of three insects, one of which, *A. pisum*, is of particular interest to us. The results are shown in Table 4. Notice that the prediction is often far from being perfect for all methods; in particular, there is as usual a delicate choice to be made between sensitivity and precision, in as much as we are currently capable of accurately measuring the latter. The low precision for all the methods may be due to two reasons. One is that the model used for predicting (pre-)miRNAs needs refinement. The other is that the precise definition of a FP miRNA is completely dependent on the known miRNAs, which could represent just a small fraction of those that really exist.

#### D6: plant sRNAseq data

Although the approaches used to treat sRNAseq data are different between our method and MIRDEEP2, we included a comparison with the latter in terms of sensitivity and precision as concerns the sRNAseq data of *A. thaliana* (dataset D6). The computing time was also compared considering only the module MIRDEEP2.PL. The parameters given to MIRINHO were the following: energy threshold of *e*=−42.8 kcal/mol (obtained as described in Section “Setting the energy threshold” using the genome of *A. lyrata*); stem length of 48nt (mean of the stem lengths in *A. thaliana*); and loop range from 5nt to 70nt (see Additional file 1: Figure S10). For MIRDEEP2, the following files were given as input: one containing the pre-miRNA and mature miRNA sequences of *A. thaliana* and another with the mature miRNA sequences of *A. lyrata*.

One may notice that the sRNAseq data are obtained under a specific experimental condition and thus not necessarily all the 57 known miRNAs of chromosome 4 of *A. thaliana* may be expressed. In order to obtain a more accurate control set, we first mapped the sRNAseq reads against the known pre-miRNAs of *A. thaliana*, and used as a control only the pre-miRNAs with a mapped read (i.e. pre-miRNAs that were expressed under the given condition). From the 57 miRNAs, 23 miRNAs were being expressed and were then used as a control. MIRDEEP2 (1120.904s) obtained a sensitivity and a precision of, respectively, 13% and 100%, and MIRINHO (1050.120s) of 83% and 38% for the same measures. MIRDEEP2 considers the highest local read stack, meaning that lower expressed pre-miRNAs may be disregarded. This can explain the low sensitivity of MIRDEEP2.

## Conclusion

We propose a fast and flexible method for the prediction of pre-miRNAs that uses minimal information about known pre-miRNAs. Concerning the prediction results, we obtain sensitivity, precision, and specificity values that are similar to those of the other tested methods, and in some cases even better. As concerns the quality of the predicted structures, the hairpins predicted by MIRINHO are much closer to the ones available in MIRBASE than the ones predicted by RNAFOLD and MIRNAFOLD. Moreover, when comparing the location where the miRNA aligns within the structure, RNAFOLD misplaced the miRNAs (putting them in a place different from a stem-arm) 10% of the time, while MIRINHO correctly placed them in all the cases.

Our method is faster because we employ a quadratic time complexity algorithm to predict the free energy of the hairpin, instead of the cubic algorithm which is commonly used. We are flexible in two aspects. First, as concerns the input type, we accept both whole genome sequences and sRNAseq data. Second, MIRINHO may be used for the prediction of either plant or animal pre-miRNAs requiring only a minimal adjustment (of the lengths of the stem-arm and terminal loop, and of the threshold for the free energy). Finally, the three features, plus the width of the diagonal, represent the only *a priori* knowledge we use.

## Availability

MIRINHO is available at http://mirinho.gforge.inria.fr.

## Additional files

Additional file 1
**Additional results.** Additional tables and graphs.

Additional file 2
**Secondary structures **(MIRBASE, MIRINHO, MIRNAFOLD). Secondary structures as presented in miRBase, and the ones predicted by Mirinho and miRNAFold.

Additional file 3
**Secondary structures **(RNAFOLD). Secondary structures predicted by RNAfold.
