# Supplementary material for: Mirinho: An efficient and general plant and animal pre-miRNA predictor for genomic and deep sequencing data
Source: BMC Bioinformatics. 2015 May 29;16:179. doi: 10.1186/s12859-015-0594-0 (PMC4448272; doi:10.1186/s12859-015-0594-0)
Supplement: Additional file 1 — Additional results. Additional tables and graphs. [file 12859_2015_594_MOESM1_ESM.pdf]

Supplementary data

Mirinho: An efficient and general plant and animal miRNA predictor for genomic and deep sequencing data

Susan Higashi, Cyril Fournier, Christian Gautier, Christine Gaspin and Marie-France Sagot

Table S1: Technical information of the current methods for the prediction of miRNAs. Information concerning the corresponding articles, type of software (standalone and/or webserver), and the implemented method. Apart from miRDeep2, MIReNA, and miRNAFold, all of the mentioned methods require a learning step.

**Name**	**Author / Paper**	**Type**	**Method**
CID-miRNA	Tyagi et al. 2008	Webserver	Stochastic Context Free Grammar
CSHMM	Agarwal etl al. 2010	Standalone	Context-Sensitive Hidden Markov Model
HHMMiR	Kadri et al. 2009	Standalone	Hidden Markov Model
microPred	Batuwita et al. 2009	Standalone	SVM
Mir-BAG	Jha et al. 2012	Standalone / Webserver	naïve Bayes, Best First Decision tree and SVM
MiPred	Jiang et al. 2007	Webserver	Random Forest
miRanalyser	Hackenberg et al. 2009	Webserver	Random Forest
miRDeep2	Friedlander et al. 2008	Standalone / Standalone	sRNAseq compatibility with Dicer processing
MIReNA	Mathelier et al. 2010	Standalone	Filtering approach
miRFinder.4.0	Huang et al. 2007	Standalone	SVM
miRNAFold	Tempel et al. 2012	Webserver	Alignment+Filtering approach
miRPara	Wu et al. 2011	Standalone	SVM
miRRim	Terai et al. 2007	Standalone	HMM
ProMiR	Nam et al. 2006	Webserver	Probabilistic co-learning model
RNAmicro	Hertel et al. 2006	Standalone	SVM
Triplet-SVM	Xue et al. 2005	Standalone	SVM

Table S2: Experiment to define the most appropriate value for the parameter *dw* (diagonal width). The numbers in bold represent the values of *dw* with the best sensitivity and precision. The aligned sequences (stem arm candidates) are all 25nt long. The energy threshold used is $e = -20.6$

	**Sensitivity** (%)	**Precision** (%)	**dw**
	38.85	0.02	4
A. aegypti	38.85	0.02	5
	39.57	0.02	**6**
	48.78	0.15	4
A. psium	49.59	0.15	**5**
	49.59	0.14	6
	41.67	0.06	4
C. Quinquefasciatus	42.50	0.05	**5**
	42.50	0.05	6
	63.37	0.28	4
H. melpomene	63.37	0.27	5
	64.36	0.27	**6**
	65.38	0.01	4
N. vitripennis	69.23	0.01	**5**
	69.23	0.01	6
	43.45	0.25	4
T. castaneum	43.69	0.24	**5**
	44.42	0.23	6

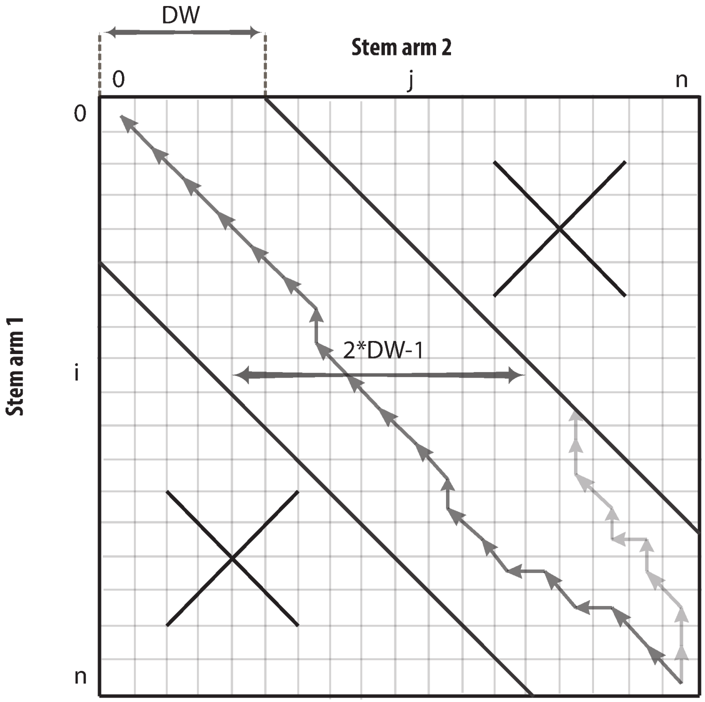

Figure S1: Illustration of the pruning strategy applied to implement the optimised version of Mirinho.

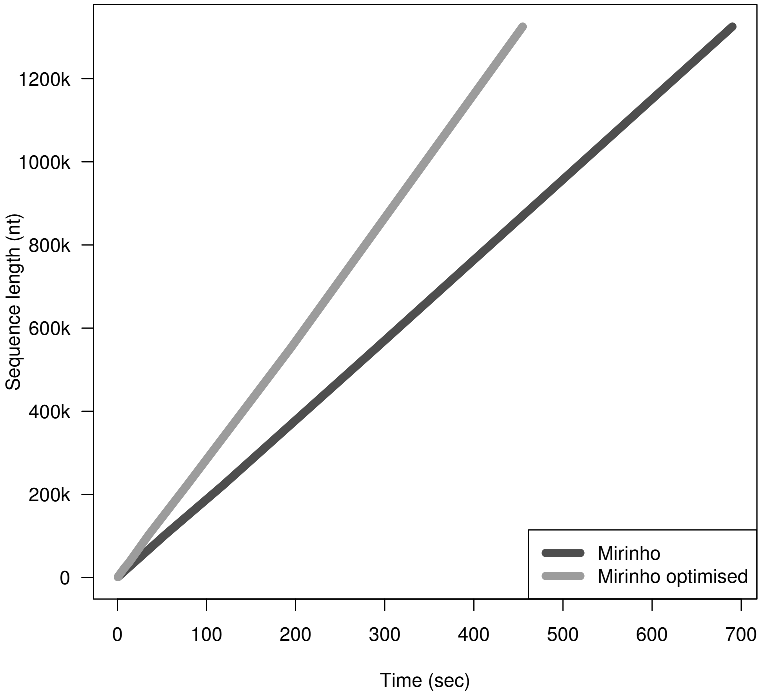

Figure S2: Time comparision between the original and the optimised versions of Mirinho. The original version consists in using the full DP matrix, while the optimised one uses only the diagonal portion of the same matrix. The times were computed on a Mac OS X Lion 10.7.5, 2.8 GHz Intel Core i7.

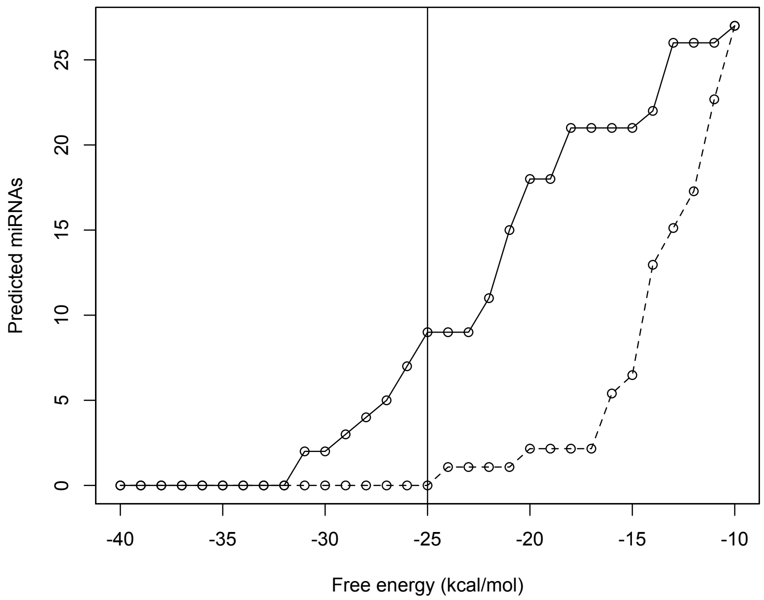

Figure S3: Number of TP miRNAs predicted when using the original and random genomes of *B. taurus*. The energy threshold is −25 kcal/mol.

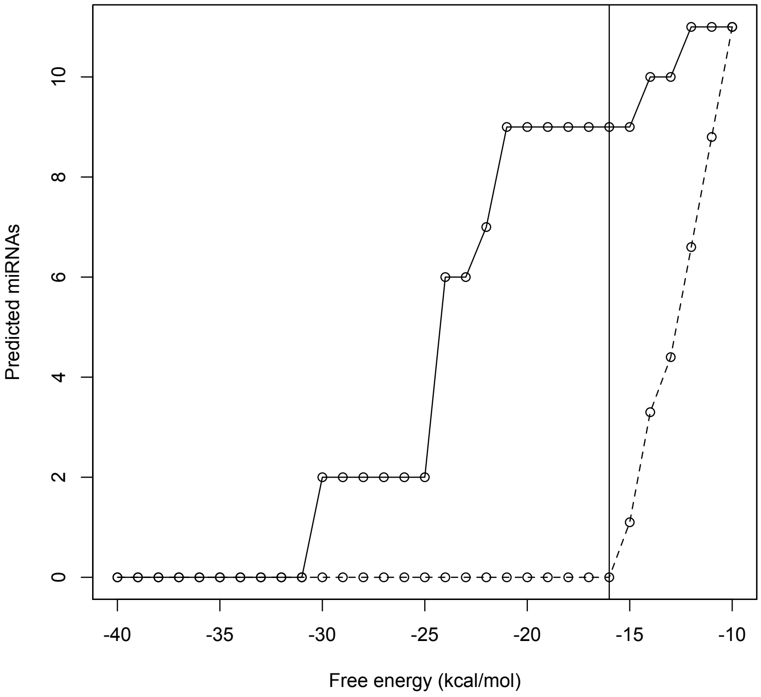

Figure S4: Number of TP miRNAs predicted when using the original and random genomes of *C. briggsae*. The energy threshold is −16 kcal/mol.

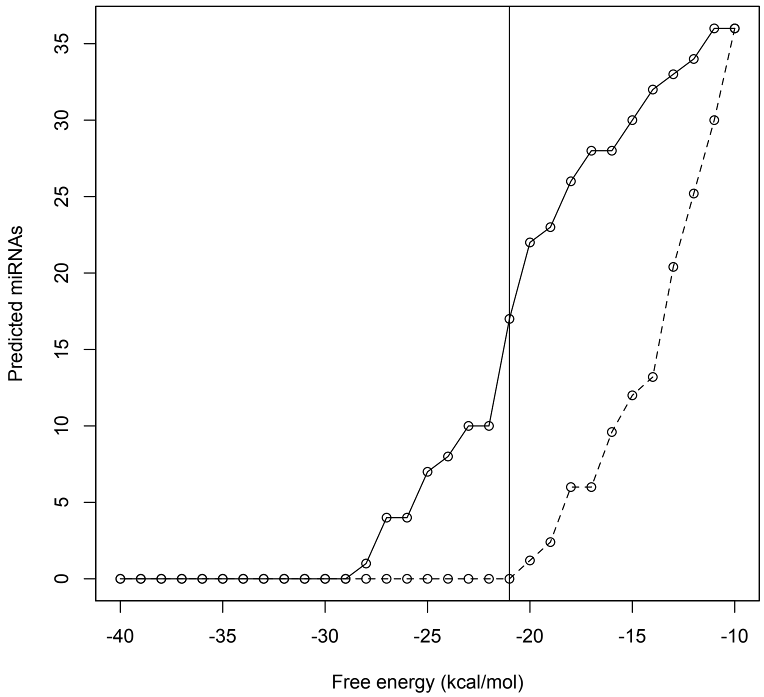

Figure S5: Number of TP miRNAs predicted when using the original and random genomes of *D. simulans*. The energy threshold is -21 kcal/mol.

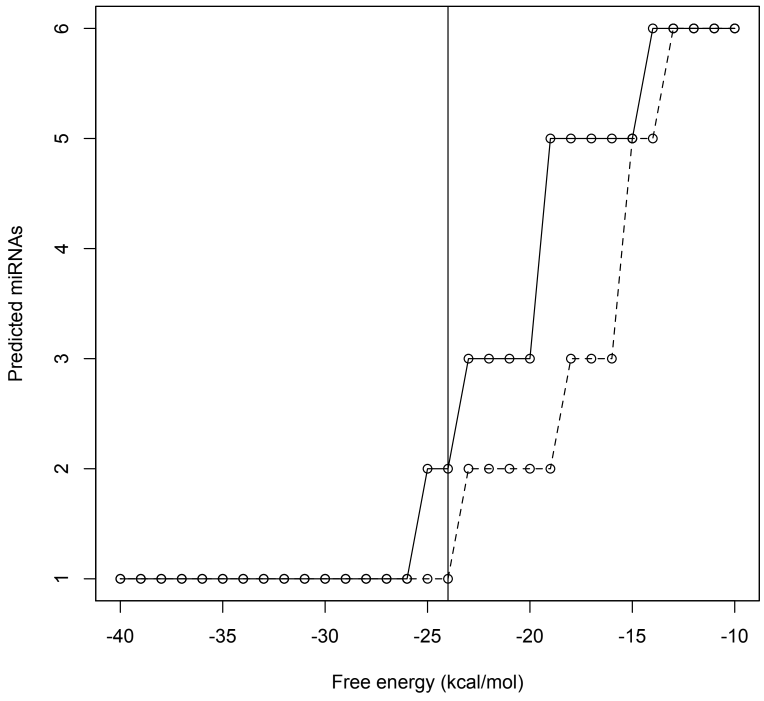

Figure S6: Number of TP miRNAs predicted when using the original and random genomes of *G. gallus*. The energy threshold is -24 kcal/mol.

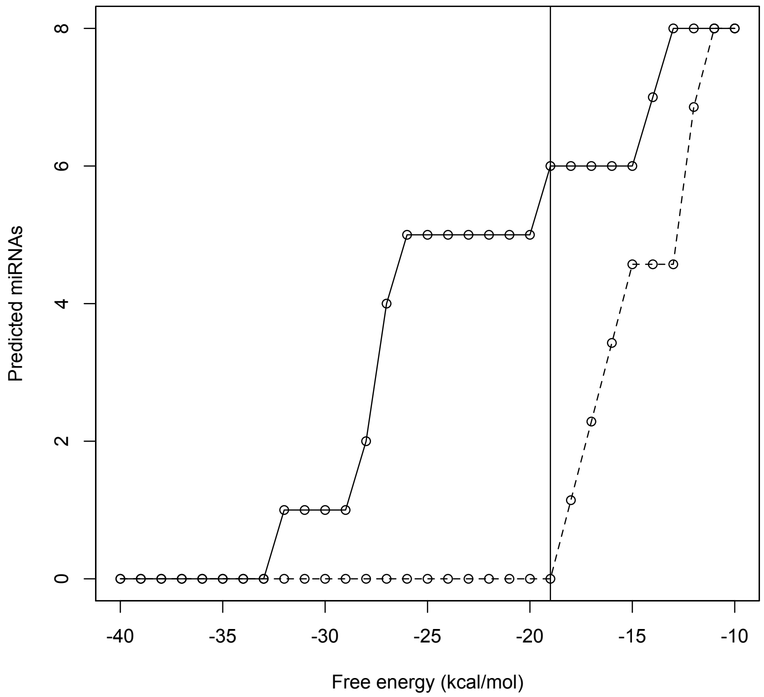

Figure S7: Number of TP miRNAs predicted when using the original and random genomes of *G. gorilla*. The energy threshold is -19 kcal/mol.

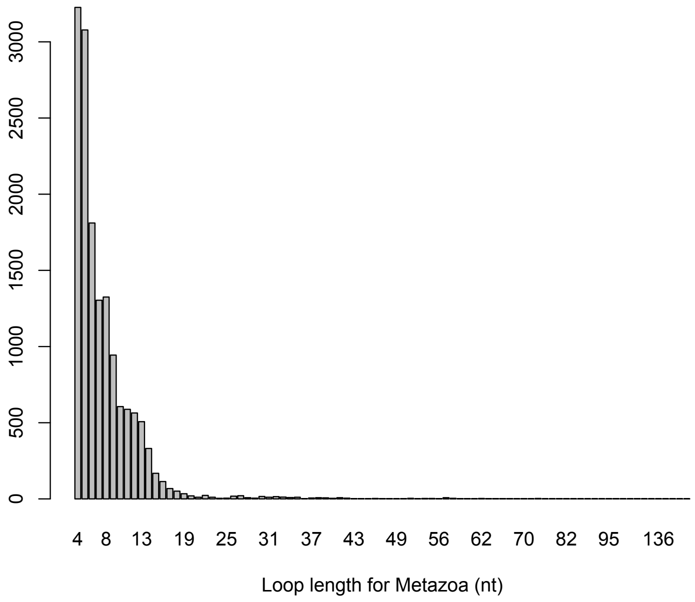

Figure S8: Terminal loop length distribution of the metazoan pre-miRNAs (miRBase release 21).
