# Supplementary material for: Mirinho: An efficient and general plant and animal pre-miRNA predictor for genomic and deep sequencing data
Source: BMC Bioinformatics. 2015 May 29;16:179. doi: 10.1186/s12859-015-0594-0 (PMC4448272; doi:10.1186/s12859-015-0594-0)
Supplement: Additional file 2 — Secondary structures (MIRBASE, MIRINHO, MIRNAFOLD). Secondary structures as presented in miRBase, and the ones predicted by Mirinho and miRNAFold. [file 12859_2015_594_MOESM2_ESM.pdf]

```
>MI0000179

-      ----           U  U  UU    -AAU   -A      ----A           -     -       AU   G   CU      U
 GCUAGA    AGAGGGAGAGA GG GA  GAGG    GCA  CAGAGA     AACUGACAGAA GAGAG UGAGCAC  GCA GCA  GUUAUG G
 ||||||    ||||||||||| || ||  ||||    |||  ||||||     ||||||||||| ||||| |||||||  ||| |||  ||||||
 CGGUUU    UCUCUCUCUCU UC CU  CUCC    CGU  GUCUCU     UUGACUGUCUU CUCUC ACUCGUG  UGU CGU  CAAUAU U
U      AAAC           C  U  CU    AGUU   CC      AUCCG           U     C       CG   G   UU      C

G     AGA        U  U  UU    A U -   -  - A A A CU A   AA  G  U   C C U CA    C  U       CU -
 CUAGA   GGGAGAGA GG GA  GAGG A G CAA CA G G A A  G CAG  GA AG GAG A A G  GGCA UG UAUGUGU  A U
 |||||   |||||||| || ||  |||| | | ||| || | | | |  | |||  || || ||| | | |  |||| || |||||||  | A
 GGUUU   CUCUCUCU UC CU  CUCU U C GUU GU C U U U  C GUU  CU UC UUU U U C  UCGU GC GUGUGCG  U A
-     AAA        C  U  CU    C C A   C  C G C C AU C   GA  G  -   C C C AC    -  -       UU C

                   -A U   -A      ----A            AGAGU   ---CA      G  ACU UUA   G
               GAGG  A GCA  CAGAGA     AACUGACAGAAG     GAG     CAUGCA GC   G   UGU U
               ||||  | |||  ||||||     ||||||||||||     |||     |||||| ||   |   |||
               CUCC  U CGU  GUCUCU     UUGACUGUCUUU     CUC     GUGCGU UG   U   AUA C
                   AG U   CC      AUCCG            ---CU   CACUC      G  CGU UCA   U

>MI0002408

             C        UU    A     U  U      ACA         A            -      UG   --U    UUCGAGCUUGGUGUGUUUUUCUAGCCAGCC
CAUUCUUAAUAUA AAUACUAC  UUUC UCCAU AA CCCCUU   AUGUCGAGU AACGAAGCAUCU GUCCCC  GUA   UGUC                              C
||||||||||||| ||||||||  |||| ||||| || ||||||   ||||||||| |||||||||||| ||||||  |||   ||||                              C
GUAAGAGUUAUAU UUAUGAUG  AAAG AGGUA UU GGGGAA   UAUAGCUCA UUGUUUUGUAGA CAGGGG  UAU   ACAG                              A
             A        UU    A     U  C      GAA         G            G      UU   UAC    UCUUAUGUUUGUUACUAGUUGAGCUCUUGA

            C        UU    A     U  U      ACA         A           -       U    U   CU CGAGCUUGGUGUGUUUUUCUAGCCAGCCCC
AUUCUUAAUAUA AAUACUAC  UUUC UCCAU AA CCCCUU   AUGUCGAGU AACGAAGCAUC UGUCCCC GGUA UGU  U                              A
|||||||||||| ||||||||  |||| ||||| || ||||||   ||||||||| ||||||||||| ||||||| |||| |||  |
UAAGAGUUAUAU UUAUGAUG  AAAG AGGUA UU GGGGAA   UAUAGCUCA UUGUUUUGUAG GCAGGGG UUAU ACA  A                              A
            A        UU    A     U  C      GAA         G           A       U    U   -C GUCUUAUGUUUGUUACUAGUUGAGCUCUUG

                        A            -      U    --U    ----UU     UUG   UG UU   U  CCA
               AUGUCGAGU AACGAAGCAUCU GUCCCC GGUA   UGUC      CGAGC   GUG  U  UUC AG   GCCC
               ||||||||| |||||||||||| |||||| ||||   ||||      |||||   |||  |  ||| ||   ||| C
               UAUAGCUCA UUGUUUUGUAGA CAGGGG UUAU   ACAG      GUUUG   UAC  G  GAG UC   UGAA
                        G            G      U    UAC    UCUUAU     --U   UA UU   C  --U

>MI0002409

       A     C      U UU    A G   U  G      ACA         A       C    -    G U        CU  G  CUU     U
CAUUCUU AUAUA AAUACU C  UUUC U CAU AA CCCCUU   AUGUCGAGU AACAAAG AUGU GUCC C AAUAUUGU  UC AG   GGUAU U
||||||| ||||| |||||| |  |||| | ||| || ||||||   ||||||||| ||||||| |||| |||| | ||||||||  || ||   |||||
```

```
GUAAGAA UAUAU UUAUGA G  AAAG A GUG UU GGGGAA   UAUAGCUCA UUGUUUU UACA CAGG G UUAUGGCA  AG UC   UUAUG U
       C     A      U UU    A G   U  G      GAA         G       C    G    G U        -U  -  ---     U

C      A     C      U UU    A G   U  G      ACA         A       C   -     G U        C  C   CU   U
 AUUCUU AUAUA AAUACU C  UUUC U CAU AA CCCCUU   AUGUCGAGU AACAAAG AUG UGUCC C AAUAUUGU UU GAG  UGG A
 |||||| ||||| |||||| |  |||| | ||| || ||||||   ||||||||| ||||||| ||| ||||| | |||||||| || |||  ||| U
 UAAGAA UAUAU UUAUGA G  AAAG A GUG UU GGGGAA   UAUAGCUCA UUGUUUU UAC GCAGG G UUAUGGCA AG CUU  GUU U
-      C     A      U UU    A G   U  G      GAA         G       C   A     G U        U  U   AU   U

                         A -A         AA       -AUG     G U        CU   AGCUU     U
               UAAGCCCCUU C  AUGUCGAGU  ACAAAGC    UGUCC C AAUAUUGU  UCG     GGUAU U
               |||||||||| |  |||||||||  |||||||    ||||| | ||||||||  |||     |||||
               GUUUGGGGGA G  UAUAGCUCA  UGUUUUC    GCAGG G UUAUGGCA  AGU     UUAUG U
                         A AA         GU       UACA     G U        -U   ----C     U

>MI0005382

AGAGACGUGGAUGCUUCUCGUCCACAAGUUCUUU   -  CUC     U      C                   --------     U  UA
                                  GGU GC   UAUGA AUAGUC GGUUUUGGAUACGUGAAAA        UCAUA CA  U
                                  ||| ||   ||||| |||||| |||||||||||||||||||        ||||| ||
                                  UCG CG   AUACU UAUUAG CCAAAACCUGUGCACUUUU        AGUGU GU  U
CAUUCUCAUCGUCCUUUUUCUACUAUUCUAUGUG   U  -UU     C      A                   AAUUGAAA     C  CG

A    C    AU CUUCUC UCC C   U C U     C UC     U      C                   - C  A CA   UG
 GAGA GUGG  G      G   A AAG U U UGGUG C  UAUGA AUAGUC GGUUUUGGAUACGUGAAAA U AU U  UAU  C
 |||| ||||  |      |   | ||| | | ||||| |  ||||| |||||| ||||||||||||||||||| | || |  |||  U
 UUCU CAUC  C      C   U UUC A G GUCGU G  AUACU UAUUAG CCAAAACCUGUGCACUUUU A UG A  GUG  G
A    -    GU CUUUUU UAC A   U U U     C UU     C      A                   A U  A AA   UC

                    C -------C     U      C                    -C  --A     UU
               GGUGC U        UAUGA AUAGUC GGUUUUGGAUACGUGAAAAU  AU   UCAUA  G
               ||||| |        ||||| |||||| ||||||||||||||||||||  ||   |||||   C
               CUAUG G        AUACU UAUUAG CCAAAACCUGUGCACUUUUA  UG   AGUGU  U
                    U UCGUCGUU     C      A                    AU  AAA     CG

>MI0013366

             --------AGC       -       C      GAUACCAA   ---U    AAUC            -----UAUAAU     CAU
CCAUUUUCGAA           CUUGAGA GAGAGAA ACAGAC        CUU    CUUC    UCGUUGCCGCAG           UAUCU   U
|||||||||||           ||||||| ||||||| ||||||        |||    ||||    ||||||||||||           |||||
GGUAAAAGCUU           GAACUUU CUCUCUU UGUCUG        GAA    GAAG    AGCGGCGGCGUC           AUAGG   C
           CAUUCACCAAA       U       U      AAAAAUAA   CAUC    -AAC            UUCCUCUCUAU     CUC

--          UAAG   UUUCUU   A G   GA A  A        A  CU G A C  C    C CCGCCGC GAAG AGAGA AU
  AUUUUCGAAG    UGG      GAA A AGA  A AC GACUUUUU UU  U U G UU UUGU G       A    G     U  A
```

```
  ||||||||||    |||      ||| | |||  | || |||||||| ||  | | | || |||| |       |    |     |
  UAAAAGCUUU    ACU      CUU U UCU  U UG UUGAAAGA AG  A A C AA GGCG C       U    U     G  U
GG          CGGA   CUCUCU   G G   GC A  G        -  UU G G -  C    U AUAUUAA AGAG AAGGA CC

                            CAGACGAUACCA     C UCAA   ----------C        A   U  UU ---UCUC
               GAGAGAGAGAACA            ACUUU U    UCU           GUUGCCGC GUA AA  A       AUUCC
               |||||||||||||            ||||| |    |||           |||||||| ||| ||  |       |||| C
               UUCUCUCUUUUGU            UGAAA A    AGA           CAGCGGCG CGU UU  U       UAGGC
                            -----------C     A --UA   ACAUCGAAGAA        G   C  CC CUCUAUA

>MI0015819

---UC    U            C         U   C       UU    ACU   UG GCA
     UUGU UGCACAUUUUGU UUUGUUAUU UCG AUGCUCC  UGAA   UUU  U   U
     |||| |||||||||||| ||||||||| ||| |||||||  ||||   |||  |
     AAUA ACGUGUGGAACA AAGCAAUAG GGC UACGAGG  ACUU   AAA  G   G
UUUCA    U            A         C   A       UC    CUC   GU UAG

-UC    U            C         U   C       UU    ACU      GCA
   UUGU UGCACAUUUUGU UUUGUUAUU UCG AUGCUCC  UGAA   UUUUGU   U
   |||| |||||||||||| ||||||||| ||| |||||||  ||||   ||||||   G
   AAUA ACGUGUGGAACA AAGCAAUAG GGC UACGAGG  ACUU   AAAGUG   G
UCA    U            A         C   A       UC    CUC      -UA

                  U            C         U   C       UU    -AC    UGUG  U
              UUGU UGCACAUUUUGU UUUGUUAUU UCG AUGCUCC  UGAA   UUUU    CA G
              |||| |||||||||||| ||||||||| ||| |||||||  ||||   ||||    ||
              AAUA ACGUGUGGAACA AAGCAAUAG GGC UACGAGG  ACUU   AAAG    GU G
                  U            A         C   A       UC    CUC    ---U  A

>MI0015820

--CU     A C          A   C       A         -      U  C      CAU   AA      UUU      UCUGAGGAUUAUUUUUCAAGAU
    UUCGU G CAUGAAGUUA UGG UAGAGUG CUAGACCCG UAACAU AC AUAUAU   UUG  CUGAAA   GUUUGU                      A
    ||||| | |||||||||| ||| ||||||| ||||||||| |||||| || ||||||   |||  ||||||   ||||||
    GAGCA C GUACUUUAGU ACC AUCUCAC GAUCUGGGC AUUGUA UG UAUAUA   AGC  GAUUUU   CAAACA                      U
CUCC     C A          C   U       -         U      U  C      ---   --      UUU      CGUCGGUGAAUAGAAGCAAAAC

GGC             A C     A      -          A      A  G       CGC -  AAA        GCAGCCACUUAUCUUCGUUUUGAU
   UCGUGGUCAUGAA U AGUGG UAGAGU GCUAGACCCG UAACAU AC AUAUAUU   U AA   AAGUUUGU                        A
   ||||||||||||| | ||||| |||||| |||||||||| |||||| || |||||||   | ||   ||||||||                        U
   AGCAUCGGUACUU A UUACC AUCUCA UGAUCUGGGC AUUGUA UG UAUAUAG   A UU   UUUAAACA                        C
GAA             C A     G      C          -      A  G       UAA C  GAC        -AACAAGACUCCUAAUAAAAAGUU

                        -       A C      CAUUGAA       UUU      UCU AG A         CAAGAU
```

```
               CUAGACCCG UAACAUU C AUAUAU        CUGAAA   GUUUGU   G  G UUAUUUUU      A
               ||||||||| ||||||| | ||||||        ||||||   ||||||   |  | ||||||||
               GAUCUGGGC AUUGUAU G UAUAUA        GAUUUU   CAAACA   C  U AAUAGAAG      U
                        U       U C      -----AGC      UUU      CGU GG G        CAAAAC

>MI0017898

    GUUGCCGU     GUUC  -   -    U       A        UAU C       AA  C    C    AA   -      A    C
CGUC        AACUC    GC CUG AGGA AAUGAUC AUUGGGUU   G UAGAGUU  AU GGGU UUGA  AGA GAAGGA GUUG A
||||        |||||    || ||| |||| ||||||| ||||||||   | |||||||  || |||| ||||  ||| |||||| ||||
GUAG        UUGAG    CG GAC UCUU UUACUAG UAACCCAA   C AUCUCAA  UA CCCA GACU  UCU UUUCCU CAGC U
    -AGUUAUU     --AU  A   A    U       C        CUU A       CC  U    C    CC   A      -    G

-A C CAA AA     UAG U  GU  A A                G A             A    GC        U  A     C C
  U U   U  AACUC   C CU  AG A AAUGAUCGAUUGGGUU A GUUAGAGUUGGAU GGGU  UGAGGAGA AA GGAGU  G A
  | |   |  |||||   | ||  || | |||||||||||||||| | ||||||||||||| ||||  |||||||| || |||||  | U
  G A   G  UUGAG   G GG  UC U UUACUAGUUAACCCAA U CGAUCUCAAUUUA CCCA  ACUUUUCU UU CUUCA  C G
CA C ACG CA     CAA C  AC  C A                A A             G    GA        C  C     A -


                   U       A        UAU C       AA  C    C    AA  -A      --A  U
               AGGA AAUGAUC AUUGGGUU   G UAGAGUU  AU GGGU UUGA  AG  GAAGGA   GU G
               |||| ||||||| ||||||||   | |||||||  || |||| ||||  ||  ||||||   ||  C
               UCUU UUACUAG UAACCCAA   C AUCUCAA  UA CCCA GACU  UC  UUUCCU   CG A
                   U       C        CUU A       CC  U    C    CC  UA      CAG  U

>MI0019217

A                  C     G           A         C        A                  U           GUAACUAACAACUGUUUUUGUCCUUAAACCA
 GUUGACUUCUAAUUUAAC UAUAA UUAUCGUUGAC CGGCCAAAU GACUCACC UUAACACUUCUUAACAGC CUCCUAACAGC                               A
 |||||||||||||||||| ||||| ||||||||||| ||||||||| |||||||| |||||||||||||||||| |||||||||||
 CAACUGAAGAUUAAAUUG AUAUU AAUAGCAACUG GUCGGUUUA CUGAGUGG AAUUGUGAGGAAUUGUCG GAGGAUUGUCG                               C
A                  U     A           G         U        C                  C           GCAAUGUUUUGUACCGGAUUGUCGGCAAUAG

A                  C     G           A         C        A                  U          - GUAACUAACAACUGUUUUUGUCCUUAAACC
 GUUGACUUCUAAUUUAAC UAUAA UUAUCGUUGAC CGGCCAAAU GACUCACC UUAACACUUCUUAACAGC CUCCUAACAG C                              A
 |||||||||||||||||| ||||| ||||||||||| ||||||||| |||||||| |||||||||||||||||| |||||||||| |                              A
 CAACUGAAGAUUAAAUUG AUAUU AAUAGCAACUG GUCGGUUUA CUGAGUGG AAUUGUGAGGAAUUGUCG GAGGAUUGUC G                              C
-                  U     A           G         U        C                  C          G CAAUGUUUUGUACCGGAUUGUCGGCAAUAG

                       A                  U           -GUA  -U    ACU    --UU   C   AAC
               GACUCACC UUAACACUUCUUAACAGC CUCCUAACAGC    AC   AACA   GUUU    GUC UUA   C
               |||||||| |||||||||||||||||| |||||||||||    ||   ||||   ||||    ||| |||    A
               CUGAGUGG AAUUGUGAGGAAUUGUCG GAGGAUUGUCG    UG   UUGU   CGGA    CGG AAU   A
                       C                  C           GCAA  UU    -AC    UUGU   C   AGC
```

```
>MI0019239

U      A                     A             U           AUAACUAACAACUGUUUUCGUCCUUAAACCA
 UGACCC GCCAAAUCGACUCACCGUUAA ACUUCUUAACAGC CUCCUAACAGC                               A
 |||||| ||||||||||||||||||||| ||||||||||||| |||||||||||                               
 ACUGGG CGGUUUAGCUGAGUGGCAAUU UGAGGAAUUGUCG GAGGAUUGUCG                               C
A      C                     G             C           GCAAUGUUUUGUACCGGAUUGUCGACAAUAG

U      A                     A             U          - AUAACUAACAACUGUUUUCGUCCUUAAACC
 UGACCC GCCAAAUCGACUCACCGUUAA ACUUCUUAACAGC CUCCUAACAG C                              A
 |||||| ||||||||||||||||||||| ||||||||||||| |||||||||| |                              A
 ACUGGG CGGUUUAGCUGAGUGGCAAUU UGAGGAAUUGUCG GAGGAUUGUC G                              C
-      C                     G             C          G CAAUGUUUUGUACCGGAUUGUCGACAAUAG

                                   A             U           -AUA  -U    ACU    --UC  CC   AAC
              GCCAAAUCGACUCACCGUUAA ACUUCUUAACAGC CUCCUAACAGC    AC  AACA   GUUU    GU  UUA   C
              ||||||||||||||||||||| ||||||||||||| |||||||||||    ||  ||||   ||||    ||  |||    A
              CGGUUUAGCUGAGUGGCAAUU UGAGGAAUUGUCG GAGGAUUGUCG    UG  UUGU   CGGA    CG  AAU   A
                                   G             C           GCAA  UU    -AC    UUGU  AC   AGC
```
