# Supplementary figures and images for: Mirinho: An efficient and general plant and animal pre-miRNA predictor for genomic and deep sequencing data
Source: BMC Bioinformatics. 2015 May 29;16:179. doi: 10.1186/s12859-015-0594-0 (PMC4448272; doi:10.1186/s12859-015-0594-0)

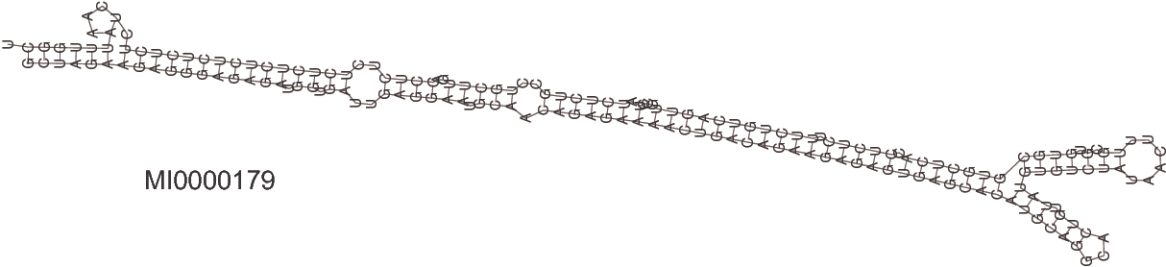

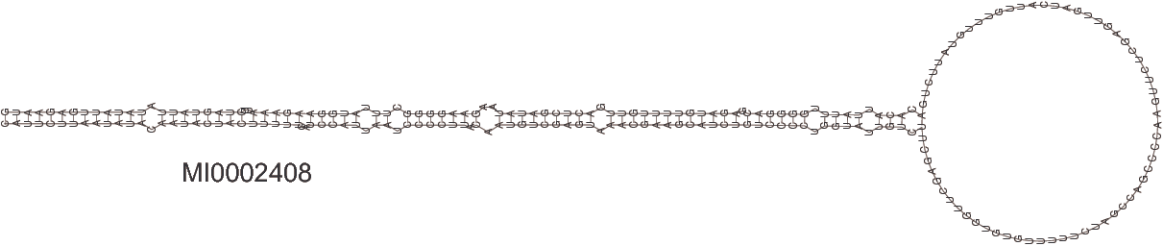

MI0002409

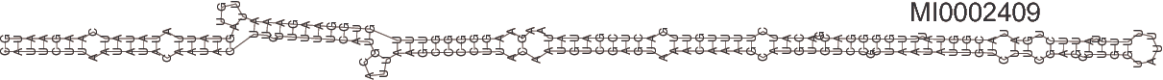

[illegible]

MI0013366

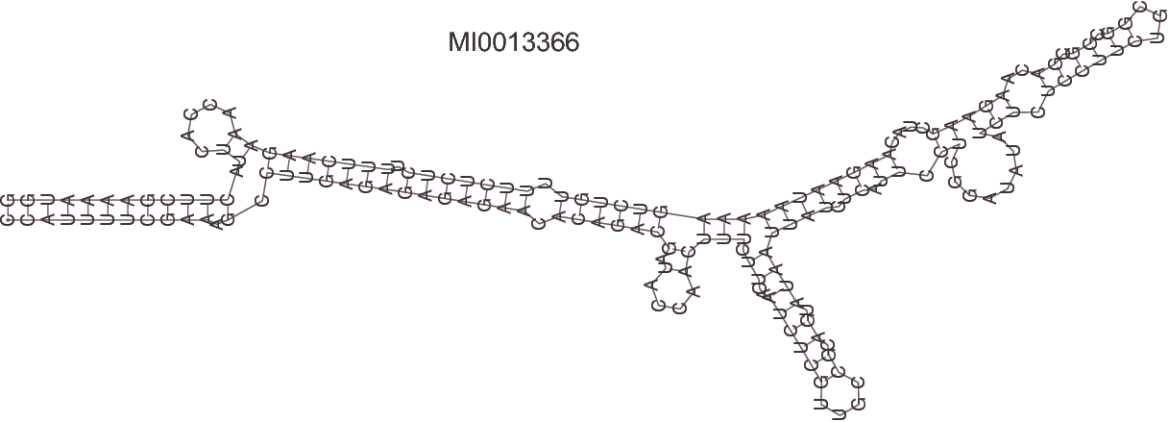

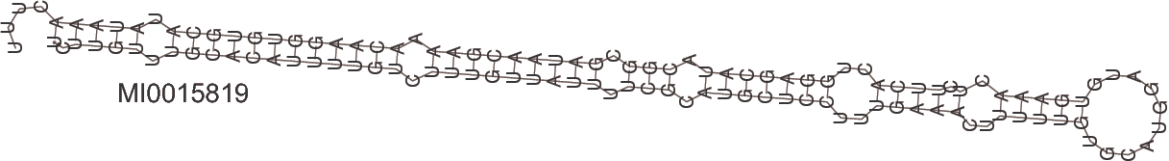

MI0015819

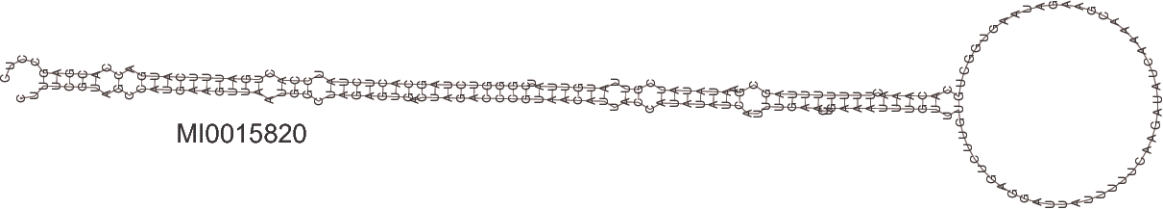

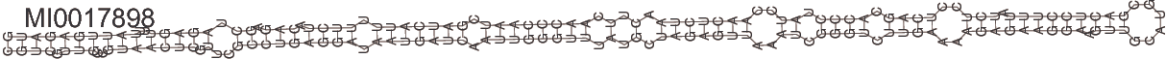

[illegible]

MI0019239

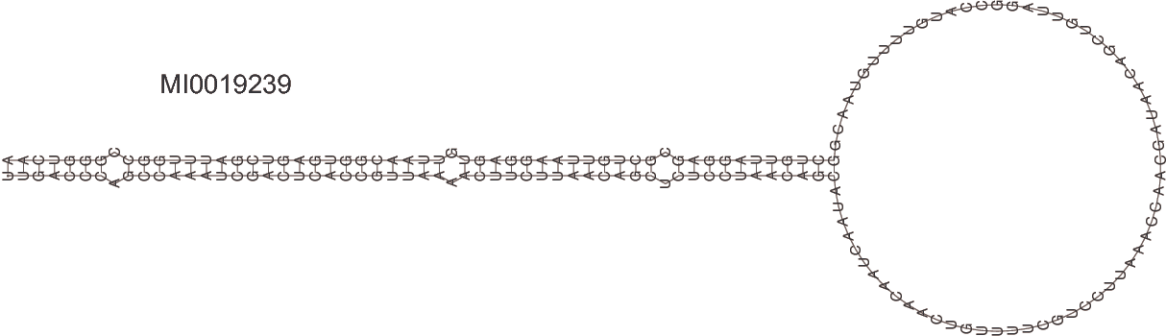

Supplement: Additional file 3 — Secondary structures (RNAFOLD). Secondary structures predicted by RNAfold. [file 12859_2015_594_MOESM3_ESM.pdf]
